# Growing pains in Danish preschool children: a descriptive study

**DOI:** 10.1038/s41598-024-54570-3

**Published:** 2024-02-17

**Authors:** Lise Hestbæk, Amanda Lücking, Sarah Thurøe Jensen

**Affiliations:** 1 Department of Sports Science and Clinical Biomechanics, University of Southern Denmark, Odense, Denmark; 2 The Chiropractic Knowledge Hub, Odense, Denmark

**Keywords:** Diagnosis, Paediatrics

## Abstract

This study provides a detailed description of growing pains in young Danish children as standardized diagnostic criteria are needed to avoid misclassifications of other musculoskeletal diagnoses. The study is nested in a cohort study of Danish preschool children. At baseline, parents completed a questionnaire with sociodemographic information. During the study, the parents received a text message every two weeks inquiring about musculoskeletal pain in the child. If pain was reported, a telephone interview about pain characteristics was conducted. The present study includes data from 2016 to 2019 with 777 children, aged 3–6 years of age at baseline. The prevalence of growing pains was 24–43%, depending on the definition. The pain occurred most frequently 1–3 times per week and most commonly in the lower legs, could be unilateral or bilateral and was usually without consequences. The prevalence increased with age, and there were no consistent associations with socio-economic factors. We suggest using Evan’s criteria with the addition of unilateral pain as standard diagnostic criteria in the future. We found no relation to periods of rapid growth and suggest that the term is a misnomer. Etiology and long-term courses of pain need to be explored in future studies.

## Introduction

Many children are affected by growing pains, which are considered a common benign syndrome without a clear pathophysiology or etiology^1–3^. The term ‘growing pains’ has been used for decades, first mentioned by the French physician Marcel Duchamp in his book “Maladies de la Croissance” from 1823^4^. As the name suggest, growing pains have been suspected of being related to growth in childhood, but to our knowledge, only one study has investigated such a link and did not find an association^5^. Further, in a recent review of 145 studies attempting to define growing pains, 93% did not refer to growth^1^ and the term has previously been called a misnomer^6,7^. Many other theories about etiology for growing pains exist, including anatomical^8^, psychological^9^, metabolic and vascular^10^, as well as Vitamin D deficiency^11–13^. Finally, the fatigue theory proposes that an increase in physical activity leads to growing pains caused by both muscular and skeletal fatigue^14^. Furthermore, some studies propose a relation to restless legs syndrome^13,15,16^. However, all of these theories are speculations as none of them has been substantiated by research^1,8,10,11^.

There is presently no conclusive definition of growing pains although the diagnosis is widely used^1^, and the condition is primarily diagnosed by exclusion, when there is no other explanation for the pain and no positive findings from the physical examination^8,17,18^. Thus, clinical signs of articular disease with articular pain, edema, redness, warmth, reduced joint range, limping or limited mobility are incompatible with a diagnosis of growing pains^8^. This is consistent with the ICD-10 diagnostic system which lists ‘growing pains’ under the diagnosis “Other symptoms and signs involving the musculoskeletal system”^19^, but interestingly, growing pains is not included in ICD-11^20^.

In 2004, Evans estimated the prevalence rate of growing pains among Australian 4–6 year-olds to be 37%^8^, but reported prevalence rates of growing pains in children to vary considerably, ranging from 3^21^ to 49%^22^. This wide range reflects a striking inconsistency in the definitions of growing pains^1^, demonstrating a strong need for a clear definition, and consensus about standardized diagnostic criteria to avoid misdiagnoses and the inherent risk of overlooking more serious and potentially treatable musculoskeletal conditions^23^.

A commonly used definition was created by Peterson^17^ and modified by Evans^8^. However, there is still conflicting opinions about the pain location. Location has been described both as bilateral pain in the lower limbs^3,8,11–13,15,16^, and as unilateral pain in the lower limbs^2^, whereas many do not specify whether it is uni- or bilateral^7,21,24,25^.

Thus, the diagnosis is still uncertain, resulting in a large risk of misclassification, which could leave other, potentially treatable, musculoskeletal problems in childhood ignored. The present study takes advantage of data from a large Danish cohort study to provide a detailed description of growing pains in Danish preschool children, with the aim to suggest standardized diagnostic criteria.

The specific objectives are:

1. To report the prevalence of growing pains.
2. To describe the clinical presentation of growing pains.
3. To describe the sociodemographic presentation of children with growing pains.
4. To investigate whether growing pains are related to rapid growth.

## Material and method

### Setting and study population

The Motor skills in Preschool (MiPS) study is an ongoing cohort study, investigating children’s musculoskeletal health, conducted by the Municipality of Svendborg and the University of Southern Denmark.

All children attending public preschools (3–6 years of age) in the Municipality (84% of the population in the age group) were invited to join the study in 2016. At baseline, the parents completed a questionnaire addressing demographic and socioeconomic factors, health status, and more. The children were tested physically, including anthropometry, by trained research staff at baseline, and at 6-, 18- and 30-months follow-up.

The MiPS study is described in detail elsewhere^26^.

### Data collection

The current study is based on data collected from September 2016, where the children were 3–6 years of age, until July 2019, where the children were 6–9 years of age.

#### Text message track

To investigate musculoskeletal complaints in the MiPS study, the parents received a text message every other Sunday. The first question was “Did [child’s name] have had any musculoskeletal pain during the past two weeks?”. In case of a positive response, the parents were interviewed by telephone within the following three days by a chiropractor to describe the complaint.

#### Telephone interview

The telephone interviews followed a structured interview guide, including questions relating to localization, frequency, diurnal fluctuations, worsening factors, perceived cause, swelling, erythema, tenderness, local trauma or infection, reduced movement and possible limping as well as consequences for the child. It was possible to report pain from more than one location.

Due to surprisingly frequent reports of growing pains from the parents, the interview guide was modified in April 2017 to systematically report variables related to growing pains, and a question was added to the text message track: “Do you think it is growing pains?”. This was intended to illustrate parents’ self-diagnosis.

### Data management

#### Categorizing of growing pains

The starting point to define diagnostic criteria in the present study was Evans’ criteria modified from Peterson’s definition from 1986^8,17^. These correspond fairly well with the most commonly reported criteria across the literature^1^ and are presented in Table 1.

**Table 1:** Evans’ diagnostic criteria^8^, modified after Peterson^17^.

Inclusion criteria	Exclusion criteria
Intermittent pain, some pain-free days and nights Bilateral leg pain (anterior thigh, calf, posterior knee-in muscles) Pain late afternoon or evening Normal physical examination No laboratory findings	Persistent pain, increasing intensity Unilateral leg pain Joint pain Pain present next morning Positive findings on physical examination: swelling, erythema, tenderness, local trauma or infection, reduced joint range of motion, limping Positive findings in laboratory tests: objective finding eg. blood test, radiograph, bone scan

However, due to conflicting opinions in the literature about localization^1^, we decided to include both unilateral and bilateral pain, as well as pain in both upper and lower extremities, and thus our five diagnostic criteria are:

- Intermittent pain with periods of days, weeks, or months without pain.
- Unexplained pain in the upper or lower extremities, uni- or bilateral, with non-articular location.
- Pain typically occurs at the end of the day or during the night, and is not present in the morning.
- No notable functional limitation or limping.
- No trauma, edema, redness, local tenderness, and no restriction in joint movement.

Based on the telephone interviews, all musculoskeletal pain descriptions were divided into three categories: “Growing pains”, “Possible growing pains” and “Not growing pains”:

*Growing pains (GP)*

The complaint fulfilled all the criteria of growing pains. ‘Intermittent’ was defined as at least 1 day, but not 7 days a week, for more than one week, and with pain free episodes. The pain was *not* considered intermittent if presence of pain free episodes could not be determined, e.g. if time of debut was missing or less than one week ago (0–7 days ago). 

*Possible growing pains (OBS-GP)*

This category represents pain that might be growing pains, but where only four of the five criteria are fulfilled or where some criteria are uncertain. This could be determined in three ways:

- The pain fulfilled four of the five criteria;
- The complaint fulfilled at least one of the criteria, whereas the rest were unclear (e.g. if the presence of pain free episodes were uncertain, as described in the definition of ‘intermittent’ above), but none were against the criteria of growing pains;
- The parents defined the complaint as growing pains, regardless of the five criteria.

*Not growing pains (Non-GP)*

Any other complaint.

#### Descriptive variables

Clinical data were extracted from the interview guide and sociodemographic data were extracted from the baseline questionnaire.

Maternal and paternal educational level were classified according to the International Standard Classification of Education (ISCED) 2011^27^. Family education was represented by the highest level of education in the family.

Equivalized disposable income was calculated as the baseline gross family income divided by an equivalence factor corresponding to the modified OECD scale^28^. The equivalized disposable income is based on gross income, available in the baseline questionnaire, instead of net income as OECD states. Thus, the absolute numbers are comparable within this study, but are not comparable to other studies. Equivalized disposable income was categorized into quartiles.

From the physical baseline test in the fall of 2016 and follow up test in the spring of 2017, we used height to calculate growth.

Details of all included variables are presented in Supplementary material Table S1.

### Data analysis

We included all children whose parents responded to the text messages at least one time during the three years (i.e. 74 text messages). To test this decision, we compared sex and baseline age between the study sample and those who responded to at least 90% of the 74 text messages, reporting means with 95% confidence intervals (CI) for age and distribution with 95% CI for sex. Further, p-values for differences between participants with more or less than 90% responses were estimated by t-test for age and Chi squared test for sex.

To test the representativeness of our study sample—both to our target population, which was all children in the municipality of Svendborg, and to the population of the entire Denmark—we made a comparison with data from children 3–5 years of age from the Danish National Statistical Database, which includes data from all citizens in Denmark^29^. We compared mean age, sex, and family constellation. Chi squared statistics were used to estimate the statistical significance of potential differences between the groups for categorical variables, and t-test for continuous variables, i.e. age.

We described the clinical presentation of growing pains using data from the first registered episode of growing pains (the index episode). Clinical characteristics were reported by numbers and percentages with 95% confidence intervals (CI).

To compare the sociodemographic profiles between growing pains groups, results were reported as proportions with 95% CI, and statistical significance was defined as no overlap between the CIs. Relative risk ratios with 95% CI were estimated using multinomial logistic regression with a separate model for each factor.

To test the theory of growing pains being related to rapid growth, we estimated a possible association between rapid growth and growing pains within the same period. As it is unknown whether absolute or relative growth is most important in relation to growing pains, we investigated the associations of both. Absolute growth was calculated as height difference from baseline to 6 months follow-up, i.e. follow up test in the spring of 2017 minus height from baseline test in the fall of 2016, and the relative growth was calculated as the absolute growth divided by the height at baseline. Rapid growth was defined as mean plus 1 SD, stratified by birth year. The index period of growing pains should be within that same period.

A potential relation between rapid growth and growing pains is presented as odds ratio based on unadjusted logistic regression analyses as we have found no evidence to suggest that a possible association could be confounded by neither age, sex nor other available factors and therefore, using a theory driven model, no confounders should be included.

STATA 17.0 was used for the analyses.

### Ethics approval

The study was approved by the Regional Committees on Health Research Ethics for Southern Denmark (S-2015-0178) as well as by the Danish Data protection Agency (2015-57-0008). This approval covered all participating centers (preschools).

Parents signed an informed consent form on behalf of the children as required by the Committee on Health Research ethics. No children will be included without written parental consent.

The study was performed according to the Declaration of Helsinki.

## Results

### Study sample

In total, 1461 children were invited to participate in the MiPS study. The parents of 8 children declined to participate and 587 never responded to the invitation. By January 1st, 2017, the cohort included 866 children.

Twelve children were excluded because their parents never responded to any of the text messages.

**Figure 1:**
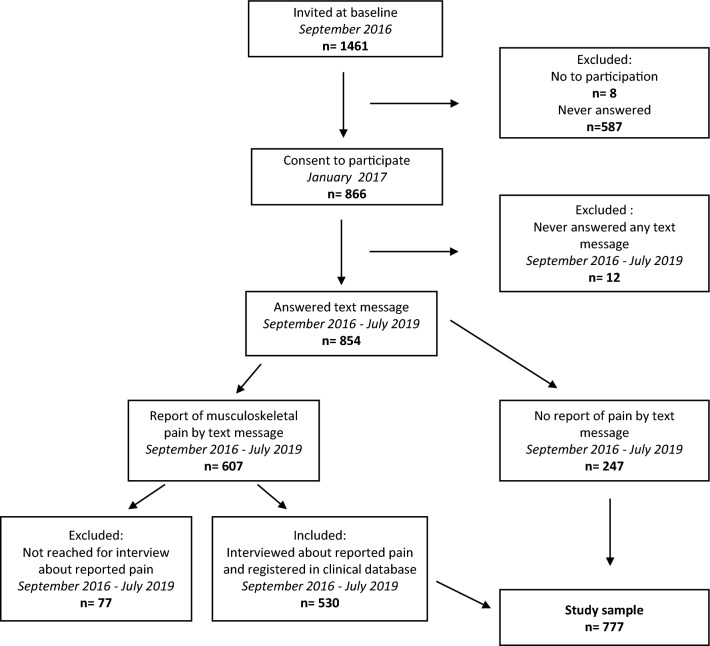
Flowchart.

During the three-year period, parents of 607 children reported musculoskeletal pain (MSK) by text message at least once. For 77 children with reports of MSK by text message (12.7%), the complaints were never described in the clinical interview database due to unanswered calls for interviews. These were excluded and the final study sample used for analyses included 777 children. A flowchart can be seen in Fig. 1.

#### Text message response rates

The mean response rate to text messages was 88% (65 out of 74 weeks) by child for the entire study period, and 40% (313 children) responded to all 74 text messages. The text message response rate was 94% in the beginning (September 2016), 89% in the middle (October 2017), and 85% at the end of the study (July 2019).

In the sensitivity analysis, including only those who responded to at least 90% of the text messages (n = 625), we had 49.1% (95% CI 45.1–53.1%) boys (n = 307), and 50.9% (95% CI 46.9–54.9%) girls (n = 318), with a mean age of 4.4 (95% CI 4.3–3.3). This is similar to the results for our analysis sample as reported in Table 2. There was no statistically significant difference between those responding to at least 90% of the text messages and those who reported less (*p* = 0.225 for sex and *p* = 0.384 for age).

**Table 2:** Representativeness of the study sample at baseline when compared to the population in the municipality of Svendborg and the entire Danish population.

	Study sample	Svendborg^a^	Denmark^b^
	*n* = *777*	*n* = *1,719*	*n* = *181,521*
**Age**,* mean (95% CI )*	4.4 (4.3–4.5)	4.1	4.02
**Sex***, %* (95% CI)	*n* = *777*	*n* = *1,719*	*n* = *181,521*
Boys	50.2 (46.7–53.7)	50.9	51.25
Girls	49.8 (46.3–53.3)	49.1	48.75
**Family constellation***, %* (95% CI)	*n* = *676*	*n* = 1773*	*n* = 186,381*
Both parents	86.1 (83.3–88.5)	79.1	81.0
Mother or father	10.7 (8.5–13.2)	16.6	15.6
Mother or father and new partner	2.8 (1.8–4.4)	3.7	2.9
Other	0.4 (0.1–1.4)	0.7	0.6
**Highest level of education in family***, %* (95% CI)	*n* = *672*		
Low, ISCED 1–2: Primary and lower secondary school	1.3 (0.7–2.6)		
Intermediate, ISCED 3–4: Upper secondary school and vocational education	23.8 (20.7–27.2)		
Academic, ISCED 5–6: Academic	48.2 (44.4–52.0)		
High academic, ISCED 7–8: High academic, 5 years +	26.6 (23.4–30.1)		

*Statistically significant difference (*p *< 0.05)  compared with our study sample.

^a^No inference measures reported as this represents the true population.

^b^Comparable data not available from Statistics Denmark. Significant values are in italics.

#### Representativeness

Essentially our baseline study sample is representative for children 3–5 years of age, living in Denmark and Svendborg, respectively, but there are a few small differences. The mean age (4.4) for our study sample is a bit higher compared to the register-based populations of Svendborg (4.1) and Denmark (4.0), respectively. The sex distribution and family constellations are similar between the three populations, but a slightly higher proportion of children in the age group live with both parents in our study sample (86.1%) than in the rest of Svendborg (79.1%) and in the entire Denmark (81.0%). Details can be seen in Table 2.

### Prevalence

During the entire study period, 530 children (68.2%) reported some kind of musculoskeletal complaint; 185 children (23.8%) were categorized as having growing pains, 148 (19.0%) as having possible growing pains, and 197 (25.3%) as having other types of musculoskeletal pain.

### Clinical presentation of growing pains

In the growing pains group, 184 children reported pain in one or more site(s) in the lower extremities, and eight in the upper extremities (it was possible to report pain in more than one location). Upper extremity pain was equally distributed with 1 or 2 reports of pain in each of the sites: shoulder, upper arm, elbow, lower arm, wrist, and hand. Only one child had pain exclusively from the upper extremity, whereas the remaining seven children also reported pain from the lower extremity. Therefore, we focused on lower extremity pain.

**Figure 2:**
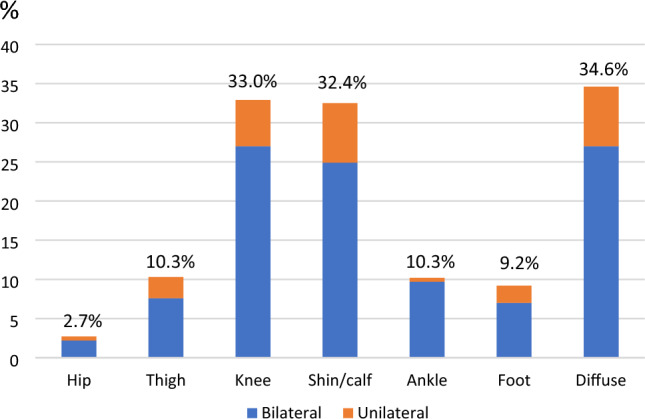
Distribution of lower extremity pain sites for children with growing pains from a Danish preschool cohort (more than one option allowed).

It was common to report pain from more than one location, as 26.1% of the 184 children reported pain from two or more locations. The most frequent pain sites n the lower extremity were ‘diffuse’ (34.6%), around the knee (33.0%), and shin/calf (32.4%), and the least frequent was the hip (2.7%). For all pain sites in the lower extremity, unilateral pain was present, but bilateral pain was more frequent (80%), with unilateral:bilateral ratios ranging from 1:18 for ankle to 1:3 for thigh pain. The distribution of pain sites in the lower extremity for the growing pains group is shown in Fig. 2, and all details can be seen in Supplementary material Table S2 for both the growing pains group and the possible growing pains group.

For the children with growing pains, it was most common to experience growing pains 1–3 times/week (80.1%) followed by 2–3 times/month. Onset was typically in the late afternoon/evening (61.6%), but night or day were not uncommon (19.5% and 14.1% respectively). Disturbed sleep was the most frequently reported consequence (26.0%), but most children (55.7%) reported no consequences.

Results were similar in the possible growing pains group, but the patterns were less distinct. Details can be seen in Table 3.

**Table 3:** Clinical profile of children presenting with growing pains or possible growing pains in a Danish preschool cohort.

	GP group In total 185 children		OBS-GP group In total 148 children	
	n	% (CI 95%)	n	% (CI 95%)
**Frequency**				
Daily	0	0.0	9	6.1 (2.8–11.3)
4–7 times/week	5	2.7 (0.9–6.2)	3	2.1 (0.4–5.8)
1–3 times/week	137	74.1 (67.1–80.2)	67	45.3 (387.1–53.6)
2–3 times/month	27	14.6(9.8–20.5)	26	17.6 (11.8–24.7)
Less often	2	1.1 (0.1–3.9)	10	6.8 (3.3–12.1)
Mentioned once	0		9	6.1 (2.8–11.2)
Missing	14	7.6 (4.2–12.4)	24	16.2 (10.7–23.2)
**Diurnal fluctuations **(more than one answer possible)				
Morning	1	0.5 (0.1–3.8)	2	1.4 (0.3–5.3)
Day	26	14.1 (9.7–19.9)	31	20.9 (15.1–28.3)
Late afternoon/evening	114	61.6 (54.4–68.4)	63	42.6 (34.8–50.7)
Night	36	19.5 (14.3–25.8)	27	18.2 (12.8–25.4)
Missing	21	11.4 (7.2–16.8)	32	21.6 (15.3–29.1)
*A combination*	13	7.0 (4.1–11.8)	7	4.7 (2.3–9.6)
Day + evening	4	2.2 (0.8–5.7)	3	2.0 (0.6–6.1)
Day + night	1	0.5 (0.1–3.8)	1	0.1 (0.1–4.7)
Evening + night	8	4.3 (2.2–8.4)	3	2.0 (0.6–6.1)
**Concequences ***(registered from week 18, 2017)*				
Reduced activity	16	8.6 (5.3–13.7)	8	5.4 (2.7–10.5)
Avoidance of specific movements	6	3.2 (1.5–7.1)	9	6.1 (3.2–11.3)
Disturbed sleep	48	26.0 (20.1–32.8)	29	19.6 (13.9–26.8)
None	103	55.7 (48.4–62.7)	73	49.3 (41.3–57.4)
Other	4	2.2 (0.8–5.7)	10	6.8 (3.7–12.2)

### Demographics

Slightly more boys than girls were in the growing pains group (25% vs. 23%), but the difference was not significant. A higher proportion of growing pains with increasing age (using birth year as a proxy for age) was reported with the relative risk of having growing pains in the youngest group (born 2013) being 0.57 (0.37–0.88) compared to the oldest group (born 2011). Age of onset is not reported, as the children were 3–6 years old at baseline with no prior data, and it is therefore unknown whether the first reported episode is the index episode.

High parental education seemed to increase the risk of growing pains (statistically significant for maternal education); however, the small size of the reference group should be noted as this could be a chance finding. There were no consistent patterns of differences between the growing pains and possible growing pains groups for number of siblings, family constellation or income. Details for this are presented in Table 4.

**Table 4:** Distribution of growing pain groups in a Danish preschool cohort by sociodemographic factors and the relative risk ratio for reporting growing pains (GP) or possible growing pains (OBS-GP) in dependence of the same factors.

	GP		OBS-GP		Non-GP
	Proportion (95% CI)	RRR (95% CI)	Proportion (95% CI)	RRR (95% CI)	Proportion (95% CI)
**Sex**	n = 185		n = 148		n = 444
Boys, n = 390	24.9 (20.8–29.4)	Ref	21.3 (17.5–25.6)	Ref	53.8 (17.5–25.6)
Girls, n = 387	22.7 (18.8–27.2)	0.81 (0.58–1.15)	16.8 (13.4–20.9)	0.70 (0.48–1.02)	60.6 (55.5–65.2)
**Birth year**	n = 185		n = 148		n = 444
2010*, n = 12	–	–	–	–	–
2011, n = 269	29.7 (24.6–35.5)	Ref	17.1 (13.0–22.1)	Ref	53.2 (47.2–59.1)
2012, n = 279	21.5 (17.1–26.7)	0.67 (0.45–1.01)	21.5 (17.1–26.7)	1.17 (0.75–1.83)	57.0 (51.1–62.7)
2013, n = 217	19.8 (15.0–25.7)	0.57 (0.37–0.88)	18.0 (13.4–23.7)	0.90 (0.55–1.46)	62.2 (55.5–68.5)
**Siblings**	n = 169		n = 135		n = 371
0, n = 83	21.7 (14.0–32.0)	Ref	32.5 (51.4–23.2)	Ref	45.8 (35.3–56.7)
1, n = 396	28.3 (24.1–32.9)	1.11 (0.61–2.03)	17.9 (14.4–22.0)	0.47 (0.27–0.82)	53.8 (48.8–58.7)
> 1, n = 196	19.9 (14.9–26.1)	0.69 (0.35–1.34)	18.9 (14.0–25.0)	0.43 (0.23–0.80)	61.2 (54.2–67.8)
**Family constellation**	n = 169		n = 135		n = 372
Both parents, n = 580	25.5 (22.1–29.2)	Ref	19.5 (16.5–22.9)	Ref	55.0 (50.9–59.0)
Mother or father, n = 70	21.4 (13.2–32.8)	0.79 (0.43–1.48)	21.4 (13.2–32.8)	1.11 (0.60–2.05)	57.1 (45.2–68.3)
Mother or father and new partner, n = 19	26.3 (10.7–51.6)	1.36 (0.44–4.21)	31.6 (14.1–56.6)	2.13 (0.72–6.27)	42.1 (21.5–65.9)
Other*, n = 7	–	–	–	–	–
**Maternal education**	n = 168		n = 133		n = 368
Low, ISCED 1–2, n = 23	8.7 (2.0–30.6)	Ref	8.7 (2.0–30.6)	Ref	82.6 (60.3–93.7)
Intermediate, ISCED 3–4, n = 92	19.6 (12.6–29.1)	4.13 (0.93–18.71)	21.7 (14.4–31.5)	3.63 (0.81–16.26)	58.7 (48.3–68.4)
Academic, ISED 5–6, n = 437	25.9 (22.0–30.2)	4.47 (1.02–19.67)	20.8 (17.3–24.9)	3.70 (0.84–16.32)	52.2 (48.6–58.0)
High academic, ISCED 7–8, n = 117	29.9 (22.2–38.9)	5.36 (1.18–24.39)	17.1 (11.3–25.1)	3.06 (0.66–14.32)	53.0 (43.9–61.9)
**Paternal education**	n = 155		n = 116		n = 326
Low, ISCED 1–2, n = 38	10.5 (3.9–25.6)	Ref	26.3 (14.5–43.0)	Ref	63.2 (46.4–77.2)
Intermediate, ISCED 3–4, n = 153	26.8 (20.3–34.4)	1.55 (0.49–4.95)	16.3 (11.3–23.1)	0.38 (0.15–0.95)	56.9 (48.8–64.5)
Academic, ISED 5–6, n = 288	27.8 (22.9–33.3)	1.54 (0.48–4.94)	18.1 (14.0–23.0)	0.52 (0.21–1.30)	54.2 (48.4–59.9)
High academic, ISCED 7–8, , n = 118	25.4 (18.3–34.1)	1.65 (0.50–5.51)	24.6 (17.6–33.2)	0.71 (0.27–1.85)	50.0 (41.0–59.0)
**Equivalized disposable income**	n = 161		n = 125		n = 335
1th quartile (lowest) , n = 147	23.8 (17.6–31.4)	Ref	22.4 (164–30.0)	Ref	53.7 (45.6–61.7)
2th quartile, n = 177	27.1 (21.0–34.2)	1.19 (0.70–2.02)	21.5 (16.0–28.2)	1.00 (0.57–1.74)	51.4 (44.0–58.7)
3th quartile, n = 138	26.8 (20.0–34.9)	1.10 (0.63–1.92)	18.1 (12.5–25.5)	0.79 (0.43–1.45)	55.1 (46.7–63.2)
4th quartile (highest) , n = 159	25.8 (19.5–33.2)	1.04 (0.60–1.79)	18.2 (12.9–25.10)	0.78 (0.44–1.40)	56.0 (48.1–63.5)

ISCED 1–2: Primary and lower secondary school ISCED 3–4: Upper secondary school and vocational education ISED 5–6: Academic, ISCED 7–8: High academic, 5 years +

*Excluded due to low numbers.

### Relation to rapid growth

Analyses were conducted on 704 children from the study sample with height measurements from both baseline and 6 months follow up. Calculation of growth revealed 6 children with unrealistic growth (two with negative growth and four with > 24 cm growth in 6 months) and these were excluded. Thus, we had growth data on 698 children, of which 101 were categorized in the growing pains group and 75 in the possible growing pains group within the period for calculating rapid growth. The distribution of rapid growth in relation to growing pains group can be seen in Table 5. There was a large but not complete overlap (94% in the same groups) between the two types of rapid growth.

**Table 5:** Number of children with growing pains, possible growing pains or no growing pains by growth groups.

	GP (n = 101)	OBS-GP (n = 75)	Non-GP (n = 522)	Total (n = 698)
Absolute rapid growth	11 (11.6%)	10 (10.5%)	74 (77.8%)	95 (100%)
Absolute non-rapid growth	90 (14.9%)	65 (10.8%)	448 (74.3%)	603 (100%)
Relative rapid growth	11 (11.2%)	12 (12.2%)	75 (76.5%)	98 (100%)
Relative non-rapid growth	90 (15.0%)	63 (10.5%)	447 (74.5%)	600 (100%)

*GP* growing pains, *OBS-GP* possible growing pains, *Non-GP* no growing pains.

The estimated odds ratios for growing pains were 0.74 (95% CI 0.38–1.45) and 0.73 (95% CI 0.37–1.43), for absolute and relative rapid growth, respectively. The results do not indicate an association between rapid growth and growing pains in this study.

## Discussion

The study’s major strengths are the large size of the cohort and the detailed clinical data. The use of text message track and telephone interviews minimize the potential recall bias, as the parents were asked about their children’s pain every two weeks and were interviewed within 1–3 days after they responded to the text message. Also, the strict classification of growing pains allowed a clear comparison between the growing pains group and the possible growing pains group.

There might however be some selection bias in our sample, with the included families having higher education and more often including both parents than average Danish families. This could potentially influence the prevalence rates, but we do not believe it hampers the validity of the descriptive data and the comparative analyses.

We found a 24% prevalence rate of growing pains in this study, using the modified criteria as explained. Because all criteria had to be fulfilled for belonging to the growing pains group, 19% were classified as having possible growing pains, mainly because it was not possible to determine whether the pain was intermittent, especially before the modification of the interview questionnaire in April 2017. Therefore, the prevalence rate is likely underestimated, and the true figure between 24 and 43%.

The pain occurred most frequently 1–3 times per week and most commonly in the lower legs. Around one fourth of the children with growing pain experienced disturbed sleep, but otherwise the pain had no consequences. Thus, the children in our cohort displayed the same characteristics as frequently reported in the literature^1^, and although the phenomenon is poorly understood, it appears to be common and reproducible across countries. Thus, the criteria listed by Evans^8^ seem to be robust and to define a specific and relatively homogenous group of pain syndromes. However, our results suggest that the pain can also be unilateral, as we saw 20.4% of complaints being unilateral in this study, while fulfilling all other criteria. Our description is based on a single episode of growing pains and previous studies’ report of bilateral pain might be observed/reported over time with pain sites changing from episode to episode, i.e. over time the pain is bilateral, although single episodes might be unilateral. Therefore, considering the large amount of unilateral pain episodes in this study, we suggest that unilateral pain should not be an exclusion criterion for growing pains.

Exclusive upper extremity pain was very rare (one child), so we agree with the Evans criteria of growing pains being related to lower extremity pain.

We found that the prevalence increased with age, and with a slight overrepresentation of boys. Most literature reporting on musculoskeletal problems also finds increasing prevalence with age but they often report higher prevalence rates for girls^30–32^. However, these studies mostly involve older children than in our sample and do not focus specifically on growing pains. Unlike most other studies^1^, we also looked at socio-demographic differences between children with and without growing pains but found no consistent associations with family constellation, parental education, or income. Nevertheless, there was an increase in growing pains prevalence with increasing maternal education, but this might be due to chance, partly because the reference group was very small, and partly due to mass-significance. Thus, unlike many other pain syndromes, e.g. back pain^33^, growing pains do not appear to be more common in the lower socioeconomic classes.

Like a previous study^5^, our results indicated no relation between growing pains and rapid growth, neither absolute nor relative growth, thus disputing the theory that the pain should be related to growth. Therefore, we agree with Al-Khattat that the term “growing pains” is a misnomer^7^ and we therefore suggest using the term “recurrent pediatric limp pain (RPL)” instead to avoid confusion about etiology. Future studies should explore other etiological theories, as a better understanding of causes and triggering factors is essential for prevention and treatment. Furthermore, it is also important to investigate the long-term course of this condition to determine whether growing pains are indeed benign, or whether they could be precursors of later musculoskeletal problems, e.g. restless legs syndrome.

## Conclusion

We found a high prevalence, 24–43%, depending on the definition, of growing pains in this cohort of 777 3–9 years old children with fairly similar presentations. Standardized diagnostic criteria are needed to avoid missing specific musculoskeletal diagnoses. Our results confirmed the relevance of Evan’s criteria, but we suggest the addition of unilateral pain.

The prevalence increased slightly with age and there were no indications of increased risk of growing pains within socially vulnerable groups. We found no relation to periods of rapid growth and therefore suggest to use the term “recurrent pediatric limp pain (RPL)” instead of growing pains.

Etiology and long-term courses of pain need to be explored in future studies with consistent use of the diagnostic criteria presented in this study.

### Supplementary Information

Supplementary Information.

## Data Availability

The data that support the findings of this study are available from the corresponding author, but restrictions apply to the availability of these data, which were used under license for the current study, and so are not publicly available. Data are however available from the authors upon reasonable request and with permission of the steering group for the MiPS project.

## Abbreviations

CI: Confidence interval
GP: Growing pains
MiPS: Motor skills in preschool
MSK: Musculoskeletal
ISCED: International Standard Classification of Education
OBS-GP: Possible growing pains
OECD: Organisation for Economic Co-operation and Development
OR: Odds ratio
RRR: Relative risk ratio
