# Supplementary material for: Growing pains in Danish preschool children: a descriptive study
Source: Sci Rep. 2024 Feb 17;14:3956. doi: 10.1038/s41598-024-54570-3 (PMC10874442; doi:10.1038/s41598-024-54570-3)
Supplement: Supplementary file 1 — Supplementary Information. [file 41598_2024_54570_MOESM1_ESM.pdf]

SUPPLEMENTARY MATERIAL

Growing pains in Danish preschool children. A descriptive study.

*Lise Hestbæk[1,2], Amanda Lücking[2], Sarah Thurøe Jensen[1]

[1] Dept. of sports science and clinical biomechanics, University of Southern Denmark, Odense, Denmark

[2] The Chiropractic Knowledge Hub, Odense, Denmark

*Corresponding author: lhestbaek@kiroviden.sdu.dk

Supplementary Table S1: Description of variables

VARIABLE	DATA SOURCE	DESCRIPTION	PRESENTATION
**Background variables**			
Baseline age - Age at the day of physical baseline test	Personal identity number	Date of test minus date of birth (embedded in the number)	Age in months
Sex	Personal identity number	Embedded in the number	Male/female
Family constellation	Baseline questionnaire	Categorial	Lives with:<br>1. Both parents<br>2. Mother<br>3. Mother and new partner<br>4. Father<br>5. Father and new partner<br>6. Other
Education - Maternal and paternal education level	Baseline questionnaire	The highest parental ongoing or completed education	1. Primary and lover secondary school (ISCED 1-2)<br>2. Upper secondary and vocational education (ISCED 3-4)<br>3. Academic education (ISCED 5-6, short cycle tertiary education and bachelor)<br>4. High academic (ISCED 7-8, master or doctoral)
Number of cohabiting siblings	Baseline questionnaire	Number of children living in the household	Number
Equivalized disposable income	Baseline questionnaire	Baseline gross family income divided by an equivalence factor corresponding to the modified OECD scale, based on family composition	Increments of DKK 100,000
Rapid growth	Physical test, anthropometry from baseline and at 6 months follow-up test	*Absolute growth:* Height at 6 months follow up minus height from baseline.<br>*Relative growth*: absolute growth divided by height at baseline.	≥ 1SD above average of absolute growth: yes/no<br><br>≥ 1SD above average of relative growth: yes/no
**Growing pains**			
Pain localization	Telephone interviews.	Categorial - reported pain in upper	Number and %<br>- Shoulder

	September 2016 – July 2019	extremities and/or lower extremities and combinations of pain sites in upper or lower extremity	- upper arm<br>- elbow<br>- lower arm<br>- wrist<br>- hand<br>- hip<br>- thigh<br>- knee<br>- shin/calve<br>- ancle<br>- foot<br>- diffuse pain<br>- combinations of pain sites in OE or UE
Frequency	Telephone interviews. September 2016 – July 2019	Categorial	Number and %<br>1. Daily<br>2. 4-7 times/week<br>3. 1-3 times/week<br>4. 2-3 times/week<br>5. Less often<br>- Missing
Diurnal fluctuations	Telephone interviews. September 2016 – July 2019	Categorial and combinations	Number and % who reported pain in morning, day, late afternoon/evening, night, or a combination:<br>- day + evening<br>- day + night<br>- evening + night.
Consequences	Telephone interviews. September 2016 – July 2019	Categorial	Number and %<br>- Reduces activity, slow down<br>- Avoidance of specific activities/movements<br>- Disturbed sleep<br>- None<br>- Other

Supplementary Table S2. Distribution of pain sites in the lower extremity in children with growing pains or possible growing pains

**Pain site**	**GP group - 185 children**		**OBS-GP group - 148 children**	
	**n**	**% (CI 95%)**	**n**	**% (CI 95%)**
Total lower extremity complaints	245		198	
Unilateral	50	*20,41*	65	*32,83*
Bilateral	195	*79,59*	133	*67,17*
Hip	5	2.7 (1.1-6.4)	4	2.7 (1.0-7.0)
Unilateral	1	0.5 (0.1-3.8)	2	1.4 (0.3-5.3)
Bilateral	4	2.2 (0.8-5.7)	2	1.4 (0.3-5.3)
Thigh	19	10.3 (6.6-15.6)	24	16.2 (11.1-23.1)
Unilateral	5	2.7 (1.1-6.4)	6	4.1 (1.8-8.8)
Bilateral	14	7.6 (4.5-12.4)	18	12.2 (7.8-18.5)
Knee	61	33.0 (26.5-40.1)	58	39.2 (31.6-47.3)
Unilateral	11	5.9 (3.3-10.5)	25	16.9 (11.6-23.9)
Bilateral	50	27.0 (21.1-33.9)	33	22.3 (16.3-29.8)
Shin/calf	60	32.4 (26.0-39.6)	45	30.4 (23.5-38.3)
Unilateral	14	7.6 (4.5-12.4)	9	6.1 (3.2-11.3)
Bilateral	46	24.9 (19.1-31.6)	36	24.3 (18.0-31.9)
Ankle	19	10.3 (6.6-15.6)	13	8.8 (5.1-14.6)
Unilateral	1	0.5 (0.8-3.8)	3	2.0 (0.6-6.1)
Bilateral	18	9.7 (6.2-15.0)	10	6.8 (3.7-12.2)
Foot	17	9.2 (5.8-14.3)	13	8.8 (5.1-14.6)
Unilateral	4	2.2 (0.8-5.7)	7	4.7 (2.3-9.6)
Bilateral	13	7.0 (4.1-11.8)	6	4.1 (1.8-8.8)
Diffuse pain UE	64	34.6 (28.1-41.8)	41	27.7 (21.0-35.5)
Diffuse unilateral pain UE	14	7.6 (4.5-12.4)	13	8.8 (5.1-14.6)
Diffuse bilateral pain UE	50	27.0 (21.1-33.9)	28	18.9 (13.4-26.1)
Combinations - *amount of locations*				
1	136	73.9 (67.0-79.8)	104	71.7 (63.8-78.5)
2	38	20.7 (15.4-27.2)	30	20.7 (14.8-28.1)
>2	10	5.4 (2.9-9.8)	11	7.6 (4.2-13.2)
